# Australian Youth Mental Health and Climate Change Concern After the Black Summer Bushfires

**DOI:** 10.1007/s10393-023-01630-1

**Published:** 2023-04-28

**Authors:** Amy D. Lykins, Melissa Parsons, Belinda M. Craig, Suzanne M. Cosh, Donald W. Hine, Clara Murray

**Affiliations:** 1 School of Psychology, University of New England, Armidale, NSW 2351 Australia; 2 School of Humanities, Arts, and Social Sciences, University of New England, Armidale, NSW Australia; 3 School of Medicine, Bond University, Gold Coast, QLD Australia; 4 School of Psychology, Speech and Hearing, University of Canterbury, Christchurch, New Zealand

**Keywords:** Climate change, Young people, Mental health, Depression, Anxiety, Wildfires

## Abstract

Climate change and its effects present notable challenges for mental health, particularly for vulnerable populations, including young people. Immediately following the unprecedented Black Summer bushfire season of 2019/2020, 746 Australians (aged 16–25 years) completed measures of mental health and perceptions of climate change. Results indicated greater presentations of depression, anxiety, stress, adjustment disorder symptoms, substance abuse, and climate change distress and concern, as well as lower psychological resilience and perceived distance to climate change, in participants with direct exposure to these bushfires. Findings highlight significant vulnerabilities of concern for youth mental health as climate change advances.

Australia already has a highly variable long-term climate (King et al., 2020), but the Intergovernmental Panel on Climate Change (IPCC) has indicated that annual temperature changes have emerged in Australia above natural variability (IPCC, 2021). Residents can expect to experience an increase in climate extremes, including heat waves, drought, and heavy rainfall, creating conditions for more frequent and severe natural hazards such as bushfires, floods, and storms (IPCC, 2021). Worldwide, the evidence for increases in climate-related illnesses, premature deaths, malnutrition in all its forms, and threats to mental health and wellbeing is strong (IPCC, 2022). A wide range of climatic events and conditions, including heatwaves, storms, and bushfires, have observable and detrimental impacts on mental health (IPCC, 2022).

Researchers (e.g., Berry et al., 2010; Hayes et al., 2018) have developed models of the proposed psychological consequences of climate change, arguing that mental health can be affected in three significant ways. First, direct impacts can occur following personal exposure to an extreme weather event (e.g., bushfire, flood). Second, indirect consequences can occur via social, economic, and environmental disruptions associated with climate change (e.g., famine, conflict, displacement). Finally, basic awareness of climate change and its current and anticipated future consequences may precipitate emotional distress (e.g., eco-anxiety) even in the absence of exposure to severe weather or climatic events.

A significant amount of research (see Galea et al., 2005; Satcher et al., 2007; Wang et al., 2013 for reviews) has documented the mental health consequences of direct natural hazard exposure, including acute stress and post-traumatic stress disorders. Indirect consequences of hazard exposure (e.g., damage to or loss of homes, infrastructure or the environment; disrupted community and healthcare resources, etc.) include mood and anxiety disorders, substance use disorders, and family conflict and domestic violence (e.g., Bryant et al., 2014). We should expect to see more of these experiences with increasing natural hazard exposure in Australia as climate change advances.

One particular group of critical importance is the world’s youth. Already noted as a population likely to suffer inordinately from climate change (Sheffield & Landrigan, 2011; Xu et al., 2012), adolescents constitute a particularly vulnerable group. The vast majority of diagnosable mental illnesses tend to onset during adolescence (American Psychiatric Association, 2013), and it is a stage where one’s ability to cope can remain limited (Tanaka et al., 2016), making this an especially sensitive time of one’s life with respect to psychological wellbeing. Compounding these pre-existing vulnerabilities is general uncertainty—futures impacted by climate change they did little to contribute to but from which they will suffer the longest-term effects. Anecdotal data (e.g., participation in climate strikes and legal actions, media articles) and reports (e.g., Dooley et al., 2021) indicate that climate change is of concern for many young people; however, empirical data are only starting to emerge about the effects climate change and related events are having on this population’s mental health and wellbeing (Hickman et al., 2021; UNICEF, 2019).

Bushfire (i.e., wildfire) season in the state of New South Wales, Australia, is typically considered to start in October (spring). In 2019, following years of extreme drought, bushfires started in June, and continued perpetually until March, 2020, culminating in what has been referred to as “Black Summer” due to the severity and intensity of the bushfires that occurred during that period. During the season, 46 million acres burned across the states of Queensland, New South Wales, South Australia, and Victoria. Nearly 6000 buildings were destroyed (half of them homes), an estimated 3 billion animals were killed or displaced, and 33 people were killed (Binskin et al., 2020). An additional 417 deaths were attributed to the effects of bushfire smoke (Borchers-Arriagada et al., 2020). Emerging evidence supports the psychological impacts Black Summer had on Australia’s youth (Gunasiri et al., 2022).

To assess mental health and climate change concern following Black Summer, young Australians in New South Wales between the ages of 16 and 25 were recruited by Qualtrics™ to complete an online self-report survey about their exposure to the recent bushfires, as well as standardized questionnaires assessing a number of mental health indicators and variables related to climate change. Data collection began in early March 2020 and went for 3 months. In all, 746 participants (584 females, 152 males, 10 “other/prefer not to say”) with a mean age of 21.1 years (*SD* = 2.79) completed the online survey. Of the total sample, 137 (18.4%) self-reported “yes” to the following question: “Have you been directly affected by any of the bushfires over the past year?” which covered the Black Summer period; 622 (83.4%) reported having been exposed to bushfire smoke. Participants received a small compensation (e.g., credits toward airline miles, gift cards) for their time.

Participants completed four standardized and validated measures of mental health.^1^ The DASS-21 (Lovibond & Lovibond, 1995) assessed current levels of depression, anxiety, and stress, with higher scores indicating higher levels of these symptoms (Cronbach’s alphas = 0.91, 0.86, and 0.89, respectively). The Adjustment Disorder New Module 8 (ADNM8; Kazlauskas et al., 2018) measured adjustment disorder symptoms, with higher scores indicating a greater reported impact of recent events on respondents’ wellbeing (Cronbach’s alpha = 0.91). The UNCOPE Alcohol and Substance Abuse Screener (Hoffmann et al., 2003) assessed current substance use patterns, with scores over 2 indicating a likelihood of substance abuse (Cronbach’s alpha = 0.84). Finally, the Brief Resilience Scale (BRS; Smith et al., 2008) assessed psychological resilience, with higher scores indicating higher levels of resilience (Cronbach’s alpha = 0.77).

Participants also completed five measures assessing perceptions of climate change.^2^ The Environmental Threat Subscale of the Environmental Attitude Inventory (Milfont & Duckitt, 2010) assessed perceived threat to the environment, with higher scores indicating higher perceived threat (Cronbach’s alpha = 0.87). Three subscales of the Psychological Distance Survey (Jones et al., 2017) assessed perceived social, geographic, and temporal distance to climate change, with higher scores indicating higher psychological distance to these aspects of climate change (Cronbach’s alphas = 0.60, 0.74, and 0.80, respectively). Finally, two measures from Hine et al. (2013) assessed concern and distress associated with climate change, with higher scores indicating greater concern and distress (Cronbach’s alphas = 0.92 and 0.90, respectively). Descriptive data for these measures can be seen in Table 1, and correlations among the measures can be found in Table 2.^3^

**Table 1:** Descriptive data and group comparisons on measures of mental health and climate change.

	Scale range	Total sample (N = 746)	Bushfire exposed (n = 137)	Not bushfire exposed (n = 609)	Between-group comparison		
		Mean (*SD*)	Mean (*SD*)	Mean (*SD*)	*df*	*F*	*p*
*Mental health*							
Depression	0–21	8.41 (6.09)	9.57 (6.25)	8.15 (6.03)	1724	5.99	0.015*
Anxiety	0–21	6.49 (5.32)	8.51 (5.44)	6.04 (5.21)	1724	24.06	< 0.001*
Stress	0–21	9.04 (5.68)	10.44 (5.81)	8.73 (5.61)	1724	9.93	0.002*
Adjustment disorder	8–48	21.77 (5.80)	23.14 (5.73)	21.46 (5.78)	1707	9.12	0.003*
Substance use	0–6	1.48 (1.89)	1.85 (1.97)	1.40 (1.87)	1741	6.23	0.013*
Resilience	1–6	3.04 (0.70)	2.89 (0.65)	3.07 (0.71)	1743	7.50	0.006*
*Climate change*							
Environmental threat	7–70	53.79 (10.05)	55.07 (9.70)	53.50 (10.12)	1742	2.72	0.099
Social distance	5–25	12.94 (3.11)	12.20 (2.91)	13.10 (3.13)	1732	9.38	0.002*
Geographic distance	6–30	15.19 (4.05)	14.10 (4.16)	15.44 (3.98)	1733	12.34	< 0.001*
Temporal distance	8–40	18.79 (5.07)	17.81 (5.18)	19.01 (5.03)	1730	6.22	0.013*
Concern	7–35	27.39 (6.18)	28.38 (6.09)	27.17 (6.18)	1743	4.31	0.038*
Distress	7–42	27.90 (7.16)	29.25 (6.71)	27.59 (7.23)	1731	6.00	0.015*

^*^Denotes a significant difference between groups. Follow-up analyses of covariance controlling for age and gender identity largely produced the same substantive results.

**Table 2:** Bootstrapped Pearson’s correlations among all study variables.

	Study measure											
	Depression	Anxiety	Stress	Adjustment disorder	Substance use	Resilience	Environmental threat	Social distance	Geographic distance	Temporal distance	Concern	Distress
Depression	–											
Anxiety	0.69**	–										
Stress	0.77**	0.80**	–									
Adjustment disorder	0.56**	0.52**	0.58**	–								
Substance use	0.33**	0.31**	0.30**	0.27**	–							
Resilience	- − 0.46**	− 0.39**	− 0.43**	− 0.41**	− 0.11*	–						
Environmental threat	0.15**	.11*	0.19**	0.21**	0.10	− 0.14**	–					
Social distance	− 0.05	− 0.10	− 0.08	− 0.09	− 0.07	− 0.05	− 0.43**	–				
Geographic distance	− 0.06	− .07	− 0.13**	− 0.05	− 0.06	0.08	− 0.42**	0.45**	–			
Temporal distance	− 0.09	− 0.07	− 0.12*	− 0.13*	− 0.07	0.10	− 0.64**	0.55**	0.50**	–		
Concern	0.10	.11*	0.12*	0.16**	0.09	− 0.14**	0.65**	− 0.55**	− 0.43**	− 0.73**	–	
Distress	0.21**	0.24**	0.22**	0.25**	0.13**	− 0.20**	0.50**	− 0.48**	− 0.35**	− 0.63**	0.71**	–

^*^Denotes significant correlation at *p* < 0.01, ** denotes significant correlation at *p* < 0.001. All correlations run with 1000 bootstrapped samples.

Analyses of variance were conducted to assess group differences between self-reported direct exposure (or lack thereof) to the Black Summer bushfires (see Table 1). Results indicated that compared to participants without direct exposure to the bushfires, those who were exposed scored significantly higher on measures of depression, anxiety, stress, adjustment disorder symptoms, and substance use, and reported significantly lower levels of psychological resilience and, furthermore, compared to participants who did not experience direct exposure to the Black Summer bushfires, those who did scored significantly higher on climate change distress and concern, and perceived significantly lower social, geographic, and temporal distance to climate change. Correlations among the mental health measures were consistent with the commonly observed comorbidity of these conditions. For example, depression, anxiety, and substance use can commonly co-occur in the presence of adjustment disorder. Importantly, the measures employed in this study target specific and non-overlapping symptoms of these disorders. Correlations of the environmental and climate change measures were in expected directions (see Table 2).

The Black Summer bushfire season was unprecedented with respect to its impact on the Australian environment and its inhabitants. To our knowledge, these are the first empirical data to be published on mental health and climate change in young Australians following these catastrophic events, adding to the overall body of knowledge on climate change and mental health in a particularly vulnerable and under-investigated population. The data presented here, collected almost immediately after the bushfire season was declared closed, reveal significantly higher levels of symptoms of depression, anxiety, stress, and adjustment disorder, as well as lower psychological resilience, in participants who reported direct exposure to the bushfires of 2019/2020. Consistent with previous research on the mental health effects of natural hazard exposure (Wang et al., 2013), and even in the context of the first wave of the COVID-19 pandemic and associated lockdowns (itself a notable stressor with respect to mental health; Nearchou et al., 2020), these young Australians were showing poorer mental health and resilience than their non-exposed counterparts.

To the extent that these data can be understood within the context of direct vs. indirect models of psychological consequences, the poorer mental health evident in the direct exposure group may reflect both immediate (e.g., higher stress disorder symptoms, as expected following direct exposure) and longer-term (e.g., higher depression, anxiety, substance use as expected following displacement, property loss) impacts of these bushfires. One could argue that the non-exposed group results may reflect a mix of indirect impacts (e.g., bushfire smoke exposure) and those resulting from the general awareness of these bushfire occurring in the state over the course of 9 months, though our data cannot specifically address these distinctions.

The bushfire-exposed group also reported higher climate change-related distress and concern, and they felt that the effects of climate change were more likely to affect them or people like them, to occur nearer to them geographically, and to happen sooner in time, than did the non-exposed participants. These results indicate a higher perceived personal vulnerability to the effects of climate change in those with direct exposure to the bushfires, also consistent with some previous research (Akerlof et al., 2013; Demski et al., 2017; Reser et al., 2014). Interestingly, there was no significant difference in general perceived threat to the environment, which was high in both groups. This finding provides additional support to recent studies highlighting the perceived threat that climate change poses to this group’s future (Hickman et al., 2021). That this was true irrespective of bushfire exposure is informative.

Though consistent with much previous research, the cross-sectional nature of these data precludes claims around causality. Future research could employ different methodologies such as longitudinal data collection to assess the potential impacts of natural hazard exposure on mental health and opinions about climate change in young people. Future research could also aim for a more even gender distribution in the sample. Even still, these data provide a unique insight into the mental health of and feelings about climate change in young Australians following the worst bushfire season in history. Furthermore, they add to the growing body of literature on climate change and mental health in young people—voices that need more attention as the climate crisis progresses.

## Footnotes

1 Sample items for mental health measures: DASS-depression (e.g., “I couldn’t seem to experience any positive feeling at all”), DASS-anxiety (e.g., “I felt scared without any good reason”), DASS-stress (e.g., “I tended to over-react to situations”), ADNM-8 (e.g., “Since the stressful situation, I find it difficult to concentrate on certain things”), UNCOPE (e.g., “Have you spent more time drinking or using drugs than you intended to?”), BRS (e.g., “I tend to bounce back quickly after hard times”).

2 Sample items for climate change scales: Environmental threat (e.g., “If things continue on their present course, we will soon experience a major ecological catastrophe”), PDS-social distance (e.g., “Climate change will definitely affect me and my family”), PDS-geographic distance (e.g., “My local area is very likely to be affected by climate change”), PDS-temporal distance (e.g., “Climate change is an immediate threat that is affecting people right now”), climate change concern (e.g., I am very concerned about climate change), and climate change distress (e.g., “I experience some distress each time I see or read media coverage of the likely impacts and consequences of climate change”).

3 ^3^The measures presented here have been selected from a larger research project due to their relevance to the focus of this study.
